# Supplementary material for: Outcome of Children and Adolescents with Recurrent Classical Hodgkin Lymphoma: The Italian Experience
Source: Cancers (Basel). 2022 Mar 13;14(6):1471. doi: 10.3390/cancers14061471 (PMC8946075; doi:10.3390/cancers14061471)
Supplement: Supplementary file 1 [file cancers-14-01471-s001.zip › cancers-1614892-supplementary.pdf]

Supplemental Material

Supplemental Table S1. – Characteristics at diagnosis of 272 recurring patients with classical Hodgkin lymphoma by first-line treatment protocol.

	MH 96 (n = 82)		LH2004 (n = 190)		
**Patient Characteristics**	***n***	**%**			***p***
Gender					0.866
Male	47	57.3	111	58.4	
Female	35	42.7	79	41.6	
Age (years)					0.006
<15	65	79.3	118	62.1	
≥15	17	20.7	72	37.9	
Histology					0.002
Nodular sclerosis	66	80.5	177	93.2	
Other	16	19.5	13	6.8	
Stage					0.750
1–2	41	50.0	91	47.9	
3–4	41	50.0	99	52.1	
Symptoms					0.680
A	35	42.7	76	40.0	
B	47	57.3	114	60.0	
Bulky					0.732
No	28	34.2	69	36.3	
Yes	54	65.9	121	63.7	
Number of involved sites					0.034
1–3	27	32.9	37	19.5	
4–7	34	41.5	82	43.2	
≥8	21	25.6	71	37.4	
Extra-nodal site involvement					0.710
No	55	67.1	123	64.7	
Yes	27	32.9	67	35.3	
Treatment group					0.097
1	7	8.5	5	2.6	
2	11	13.4	24	12.6	
3	64	78.1	161	84.7	
Radiotherapy					0.001
No according to protocol	4	4.8	2	1.0	
No for disease progression	10	12.2	58	31.0	
Yes	68	82.9	127	67.9	

Extra-nodal sites: parenchymal sites including liver, lung, bone, and bone marrow. % percentages calculated on valid data only.

Supplemental Table S2. – Characteristics at the disease recurrence of 272 patients with classical Hodgkin lymphoma by first-line treatment protocol.

	MH 96 (n = 82)		LH2004 (n = 190)		
**Patient characteristics**	***n***	**%**	***n***	**%**	***p***
Type of recurrence					<0.001
Progression	21	25.6	96	50.5	
Relapse	61	74.4	94	49.5	
Early relapse	30	49.2	49	52.1	0.720
Late relapse	31	50.8	45	47.9	
Age (years)					0.002
<15	49	59.8	75	39.5	
≥15	33	40.2	115	60.5	
Stage					0.269
1–2	40	56.3	81	48.5	
3–4	31	43.7	86	51.5	
Number of involved sites*					<0.001
1	33	44.6	39	23.2	
2–4	33	44.6	69	41.1	
≥5	8	10.8	60	35.7	
Extra-nodal site involvement					0.136
No	53	71.6	103	61.7	
Yes	21	28.4	64	38.3	
Recurrence at the same site					0.006
No	20	27.0	21	12.5	
Yes	54	73.0	147	87.5	
Recurrence after radiotherapy					0.262
In non-irrad. sites	13	21.0	17	14.4	
In the irrad. site	49	79.0	101	85.6	

Extra-nodal sites: parenchymal sites including liver, lung, bone, and bone marrow. OT = Off Therapy. % = percentages calculated on valid data only. * Mean values: 2.5 (sd: 1.9) vs. 3.9 (sd: 3.1); median values 2 (IQR: 1-4) vs 3 (IQR: 2-5), $p < 0.001$.

Supplemental Table S3. – Ten-year Event Free Survival (EFS) of 272 patients with classical Hodgkin lymphoma after recurrence by characteristics at diagnosis.

**Patient Characteristics**	**N/E**	**EFS**	**95%CI**	***p***
Whole cohort	272/121	53.3	46.7–59.3	-
Protocol				0.092
MH96	82/45	45.4	34.3–55.8	
LH2004	190/76	56.5	48.1–64.1	
Gender				0.326
Male	158/74	51.0	42.5–58.9	
Female	114/47	56.4	46.2–65.5	
Age (years)				0.873
0–14	183/83	53.7	46.0–60.8	
≥15	89/38	49.9	36.8–61.7	
Histology				0.833
Lymphocyte depleted	5/2	50.0	5.8–84.5	
Mixed cellularity	24/12	54.6	32.0–71.1	
Nodular sclerosis	243/107	53.2	46.3–59.7	
Stage				0.120
1–2	132/53	58.3	49.1–66.3	
3–4	140/68	48.7	39.4–57.3	
Symptoms				0.065
A	111/42	61.6	51.6–70.1	
B	161/79	47.7	39.1–55.7	
Bulky disease				0.807
No	97/42	54.1	42.9 – 64.1	
Yes	175/79	52.9	44.9 – 60.3	
Number of involved sites *				0.342*
1–3	64/25	61.4	48.1–72.2	
4–7	116/54	50.4	40.3–59.6	
≥8	92/42	50.9	39.1–61.5	
Extra-nodal sites involvement**				0.752
No	178/80	53.3	45.4–60.7	
Yes	94/41	53.4	42.0–63.5	
Treatment group				0.240
1	12/3	74.1	39.1–90.9	
2	35/13	63.2	44.4–77.2	
3	225/105	50.6	43.4–57.4	
Radiotherapy				0.115
No according to protocol	6/1	83.3	27.3–97.5	
No for disease progression	68/35	46.1	33.3–58.0	
Yes	195/83	55.0	47.2–62.1	

*The number of involved sites was grouped according to the tertiles of its distribution. **Extra-nodal sites: parenchymal sites including liver, lung, bone, and bone marrow. % percentages calculated on valid data only.

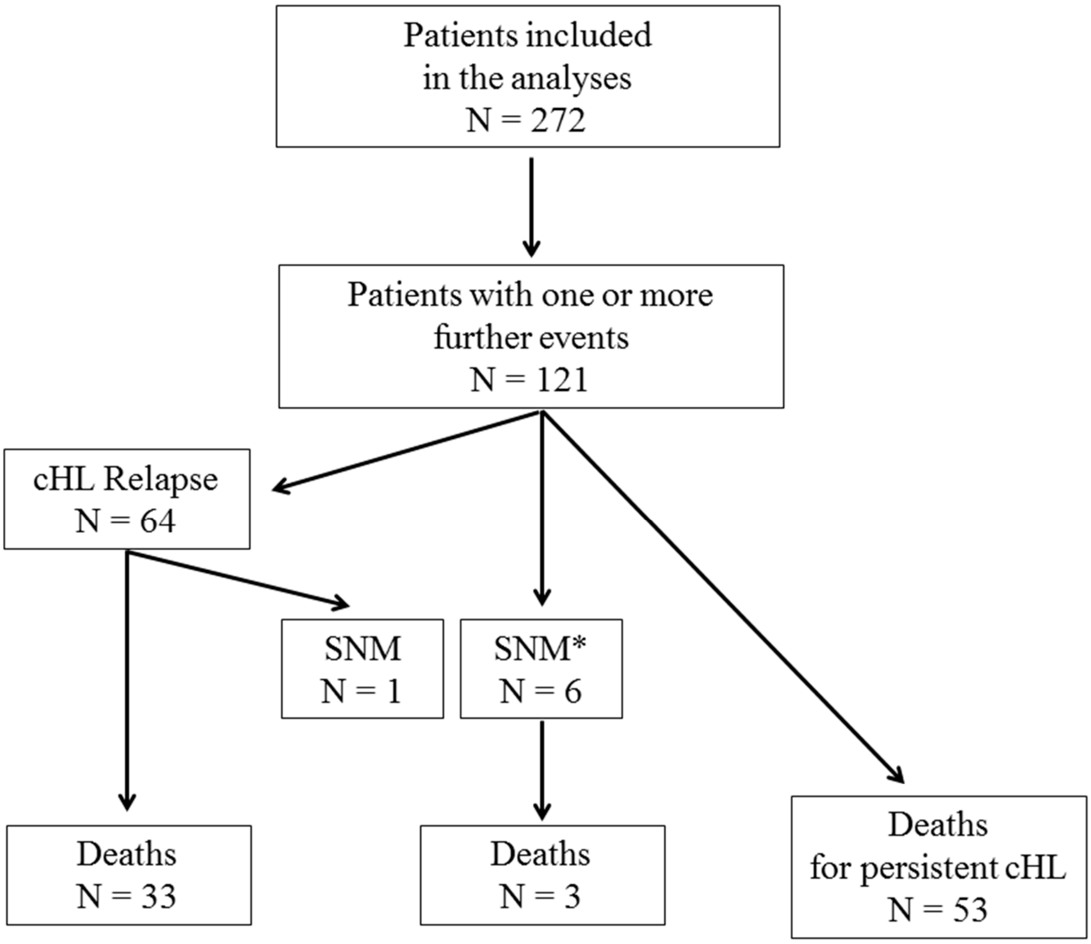

*6 SNM were observed in 4 patients: two patients had one SNM, one patient had a concurrent secondary SNM and another patient had two subsequent SNM

Supplemental Figure S1. Patients' outcome after the first disease recurrence.
